# Legal status of recreational cannabis and self-reported substitution of cannabis for opioids or prescription pain medication in Canada and the United States

**DOI:** 10.1080/08897077.2022.2060431

**Published:** 2022-01-01

**Authors:** Elle Wadsworth, Lindsey A Hines, David Hammond

**Affiliations:** 1 School of Public Health Sciences, University of Waterloo, Waterloo, Canada; 2 Population Health Sciences, Bristol Medical School, University of Bristol, Bristol, UK; 3 Integrative Epidemiology Unit, Bristol Medical School, University of Bristol, Bristol, UK

**Keywords:** opioids, pain, cannabis, marijuana, legalization, substitution

## Abstract

**Aims:**

With increased liberalization of cannabis policies in North America, there is growing interest in the use of cannabis to manage pain instead of opioids. The objectives of the study were to: 1) examine the use of cannabis for pain relief in Canada and the United States (US) in 2018 and 2019; 2) examine the association between recreational cannabis laws and changes in the use of cannabis for pain relief, instead of opioids or prescription pain medication.

**Methods:**

Repeat cross-sectional survey data were used from Wave 1 and Wave 2 of the International Cannabis Policy Study conducted in 2018 and 2019 in Canada and the US. Respondents were recruited through commercial panels, aged 16-65, and had ever tried cannabis (N=44,119). Weighted binary logistic regression models examined the association between the legal status of recreational cannabis and cannabis use for pain relief instead of opioids or prescription pain medication (n=15,092).

**Results:**

Between 14%-33% of cannabis consumers in Canada and the US reported using cannabis to manage headaches or pain. Of these consumers, 79% and 78% respondents in Canada; 80% and 83% in US illegal states; and 83% and 84% in US legal states, in 2018 and 2019, respectively, reported cannabis use for pain relief instead of opioids or prescription pain medication. There was little evidence of an association between the legal status of recreational cannabis and cannabis use for pain relief instead of opioids or prescription pain medication, among Canadian (AOR=0.98, 95% CI: 0.78, 1.22) and US respondents (AOR=1.11, 95% CI: 0.96, 1.28).

**Conclusions:**

Although substitution of cannabis for opioids or prescription pain medication is common among those who use cannabis for pain, there does not seem to be a significant difference according to cannabis legality. Future research should examine cannabis and opioid substitution using different research designs and time frames.

## Introduction

In recent years, non-medical (hereafter ‘recreational’) cannabis policy has liberalized in North America. In 2018, Canada legalized recreational cannabis. As of 2021, 18 US states and the District of Columbia (DC) have legalized or passed laws to legalize recreational cannabis, with more predicted to follow. With increased access to legal cannabis, there has been growing interest in cannabis for the management of pain instead of opioids.^1–3^ Indeed, as the same cannabis products can be used for both recreational and medical purposes, the legalization of recreational cannabis could impact the use of cannabis for pain relief. Opioid use for pain management is associated with increased risk of overdose, and there is some evidence that increased access to cannabis through legalization is linked to a reduction in opioid prescriptions and opioid-related deaths.^2,4–10^

Although literature on cannabis as an effective treatment for pain is inconclusive,^11–13^ cannabis is still widely used for pain relief.^14–18^ In studies among people who use drugs and/or experience chronic pain, there is evidence of cannabis being substituted for prescription medication or opioids.^15–18^ For example, in a cross-sectional study examining the substitution of cannabis among medical cannabis patients, 63% reported substituting prescription medication for cannabis, with opioids being the most commonly substituted medication.^17^ However, in other patient populations, such as those who use non-medical opioids, cannabis was not used as a substitute.^19,20^ In addition, the opioid-sparing effect of cannabinoids - where the use of cannabinoids requires lower doses of opioids to achieve similar levels of pain relief - is inconsistent among clinical and observational samples.^21,22^

Reviews examining the relationship between cannabis legalization and opioid use found inconclusive evidence that legalization reduced the use and harms of opioids, and that more evidence is needed to ensure associations could not be explained by other jurisdiction-level factors.^2,23–25^ For example, a recent study of the relationship between medical cannabis laws and opioid mortality concluded mortality was higher in states with medical cannabis laws but that the results could be explained by better overdose reporting in those states.^26^ In order to address such issues, there is a need to replicate studies across different contexts.

This descriptive study explored the relationship between prescription pain medication (PPM), opioids, and cannabis in North American jurisdictions with differing recreational cannabis policies, with an additional focus on the relationship between recreational cannabis laws (RCL) and substitution of cannabis for opioids or PPM. Replication can strengthen inference when factors that may lead to uncertainty are varied; in the present study, results are replicated in jurisdictions with different RCL, including pre- and post-legalization in Canada as well as US states that have (US ‘legal’ states) and have not legalized recreational cannabis (US ‘illegal’ states).

The aims of the study were to: 1) describe trends in cannabis for pain relief, by year and RCL, and test differences by year among those who have ever tried cannabis; 2) examine the association between RCL and changes in the use of cannabis for pain relief, instead of opioids or PPM, among those who have ever used cannabis for pain relief.

## Methods

### Study design and sample

Data are repeat cross-sectional findings from Waves 1 and 2 of the International Cannabis Policy Study (ICPS) conducted in Canada and the US. Data were collected via self-completed web-based surveys conducted between August-September in 2018 and September-October in 2019 with respondents aged 16-65. Respondents were recruited through the Nielsen Consumer Insights Global Panel and their partners’ panels. A full description of the study design and methodology can be found elsewhere.^27–29^

After removing respondents from DC due to insufficient sample size, and respondents from Michigan due to a change in cannabis legislation across 2018 to 2019, the cross-sectional samples comprised of 26,806 respondents in 2018 and 43,322 in 2019. The current analysis was based on the sub-sample of respondents who had ever tried cannabis (Canada: n=15,354; US: n=28,765). The sample for regression analysis was restricted to ever cannabis consumers who reported consuming cannabis for pain relief (Canada: n=4,368; US: n=10,724) (Online Supplemental Material 1).

The study was reviewed by and received ethics clearance through a University of Waterloo Research Ethics Committee (ORE#31330).

### Measures

Full item wording is available in the ICPS 2018 and 2019 survey (www.cannabisproject.ca/methods). All questions included “Don’t know” and “Refuse to answer” options. In all measures, “Refuse to answer” and “Don’t know” was treated as missing.

#### Exposure

In Canada, RCL were represented by survey year: 2018 (pre-legalization) vs. 2019 (post-legalization). In the US, RCL were represented by jurisdiction: US ‘illegal’ states vs. US ‘legal’ states. As a sensitivity analysis, US states were also categorized by medical cannabis laws: US states with recreational and medical laws vs. medical laws vs. prohibited or cannabidiol (CBD) laws.

#### Outcomes

Respondents who had ever used cannabis to improve/manage headaches or pain were asked “Have you ever used cannabis for pain relief, instead of using opioids or prescription pain medication?” (Yes/No).

#### Covariates and substance use measures

Age, sex at birth, education level, ethnicity/race, perceived income adequacy, and cannabis frequency. See Table S1 for full coding of response options.

Respondents who had ever tried cannabis were asked “Have you ever used cannabis to improve/manage symptoms for headaches/migraines” and “Have you ever used cannabis to improve/manage symptoms for pain” (Yes/No).

### Statistical analysis

Post-stratification sample weights were constructed based on the Canadian and US Census estimates. Separately for each jurisdiction, a raking algorithm was applied to the cross-sectional analytic samples to compute weights that were calibrated to these groupings. Weights were rescaled to the jurisdiction’s sample size.^27–29^ Estimates are weighted unless otherwise specified.

First, descriptive statistics were used to describe the use of cannabis for pain relief across jurisdictions in 2018 and 2019. Two-sample tests of proportions examined differences between percentages across the survey years, and were conducted using STATA/MP version 16.0. Second, binary logistic regression models were fitted to estimate univariable and multivariable estimates of the association between RCL and using cannabis for pain relief instead of opioids or PPM. As a sensitivity analysis, analyses among US respondents were repeated with medical cannabis laws as the exposure. All models were adjusted for sociodemographic characteristics and cannabis frequency. Models with US data were also adjusted for survey year (survey year is the exposure variable for Canadian data). As a sensitivity analysis to explore potential moderating and confounding effects of cannabis frequency, models were run with cannabis frequency included as a main effect, excluding cannabis frequency, and included as a two-way interaction with survey year.^30^ Unadjusted Odds Ratios (ORs) and adjusted odds ratios (AORs) are reported with 95% confidence intervals (95% CI). Analyses were conducted using survey procedures in SAS (SAS version 9.4, SAS Institute Inc., Cary, NC, US).

## Results

Online Supplemental Material 2 displays the sample characteristics among respondents in Canada, US illegal and US legal states in 2018 and 2019. Sample characteristics remained largely consistent across years except ethnicity/race and cannabis frequency.

### Changes in cannabis for pain relief across jurisdiction in 2018 and 2019

Table 1 displays the use of cannabis for pain relief across jurisdiction in 2018 and 2019. The percentage of ever cannabis consumers who reported using cannabis to improve/manage headaches or pain increased in all jurisdictions from 2018 to 2019. Among respondents who reported consuming cannabis for headache/pain management, approximately 78-85% reported to use cannabis for pain relief instead of opioids or PPM in 2018 and 2019.

Online Supplemental Material 3 displays cannabis use frequency and problematic cannabis use prevalence among ever cannabis consumers who reported using cannabis to improve/manage headaches or pain.

### Cannabis use for pain relief across legal and illegal jurisdictions

As shown in Table 2, there was little evidence of an association between RCL in Canada or the US and the use of cannabis for pain relief instead of opioids or PPM. In a supplementary analysis, there was little evidence of an association between the legal status of recreational and medical cannabis and the use of cannabis for pain relief instead of opioids or PPM (Online Supplemental Material 4).

## Discussion

The current study found that between 14% and 33% of cannabis consumers in Canada and the US reported using cannabis for pain relief, with minimal change between 2018 and 2019. Among respondents who reported consuming cannabis for pain relief, most reported substitution of cannabis for opioids or PPM, with minimal change between 2018 and 2019. There was little evidence of an association between the likelihood of substituting cannabis for opioids or PPM and either medical or recreational cannabis laws.

Most respondents who reported consuming cannabis for pain relief reported lifetime substitution of cannabis for opioids or PPM in both years. These results are similar to a cross-sectional study among Canadian medical cannabis consumers that demonstrated most consumers used cannabis in substitution for prescription drugs and those who reported pain as their main reason for cannabis use had a higher likelihood of substitution.^17^ This may suggest that consumers could be a population to benefit from cannabis for pain relief over opioids. However, the opioid-sparing effect of cannabis is not established and lifetime substitution may not result in more recent substitution; therefore, future research should continue to examine the efficacy of cannabis substitution.^21–22^

There was little evidence of an association between RCL and cannabis substitution. By replicating the models in two countries, the current study can strengthen inference that the little evidence that was found was not confounded by demographic variables or other cannabis laws. These results add to the emerging literature of legalization and its association with cannabis and opioid substitution.^2,8,22,23,31,32^ To date, the findings are mixed, with no consistent evidence of an association between cannabis legalization and opioid use or harms. Previous research in the US found an association between legalization and reduced overdose mortality between 1999 and 2010;^8^ however, studies that included additional years concluded no relationship or an inverse relationship.^22,23,31,32^ Experimental studies are needed to advance the literature and clarify the association between cannabis legalization and opioid use.

### Limitations

This study is subject to limitations common to survey research. Respondents were recruited using non-probability-based sampling; therefore, the findings do not provide nationally representative estimates. Cannabis use estimates were within the range of national estimates for young adults, but higher for the entire ICPS sample in Canada. This is likely because the ICPS do not sample individuals over 65, who are known to have lower rates of cannabis use.^27–29^

The current study uses ‘ever use’ to measure substituting cannabis for pain relief. This influences the ability to detect an effect of legalization as lifetime use would include substitution before legalization. Moreover, the current study is limited to those who had ever used cannabis for pain and so cannot be generalized to all cannabis consumers, all opioid consumers, or all PPM consumers.

Regression models were adjusted for cannabis frequency as a confounding variable, but it is plausible that cannabis frequency may act as a moderator. As a sensitivity analysis, cannabis frequency was both removed and included as an interaction with survey year, and similar patterns were observed. When cannabis frequency was included, there was strong evidence of an association between cannabis frequency and the likelihood of substitution in both Canada and the US, which complement conclusions found among medical cannabis patients and people who use drugs.^16–18^ It is plausible that the relationship between frequent cannabis consumption and opioid substitution is bi-directional: frequent cannabis consumers may be more likely to engage in substitution, and those who have substituted may be more likely to consume cannabis frequently. Moreover, frequent consumers could experience pain as a symptom within cannabis withdrawal syndrome, and use cannabis or pain medication in response. However, this study uses repeat cross-sectional data and therefore cannot determine direction nor causality.

### Conclusion

Although substitution of cannabis for opioids or PPM is common among those who use cannabis for pain, the findings demonstrate little evidence of an association between the likelihood of substituting cannabis for opioids or PPM amongst those who use cannabis and RCL. However, it is likely that the effect of increased access to legal cannabis may take considerable time to be observed at the population level, particularly given the time required to establish legal retail markets. Future research should explore the substitution of cannabis and opioids in legal markets over multiple years and among different population subgroups, including through the use of different research designs.

## Supplementary Material

Supplementary materials

## Abbreviations

US: United States
PPM: Prescription pain medication
RCL: Recreational cannabis laws
