# Supplementary material for: Legal status of recreational cannabis and self-reported substitution of cannabis for opioids or prescription pain medication in Canada and the United States
Source: Subst Abus. Author manuscript; Available in PMC 2023 Aug 14. (PMC7614954; doi:10.1080/08897077.2022.2060431)
Supplement: Supplementary materials [file EMS181576-supplement-Supplementary_materials.docx]

**Online Supplemental Material 1: Sample size flowchart for measures**

**
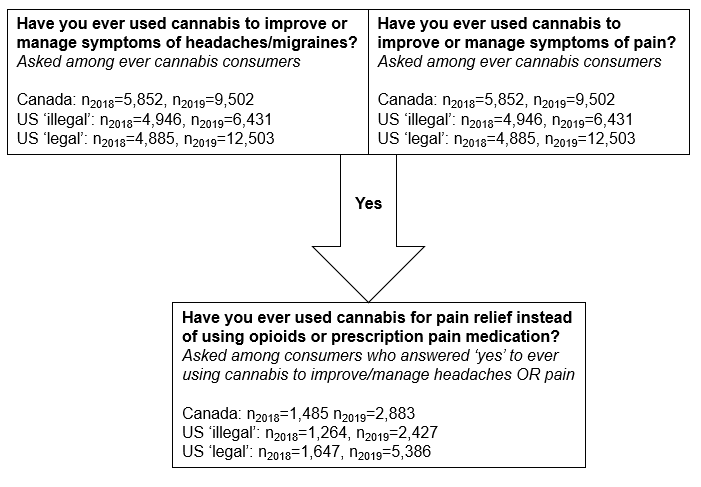
**

**Online Supplemental Material 2: Sample characteristics across Canada, US ‘illegal’ states, and US ‘legal’ states in 2018 and 2019 (n=44,119)**

|  | Canada  % (n) | | US ‘illegal’ states  % (n) | | US ‘legal’ states  % (n) | |
| --- | --- | --- | --- | --- | --- | --- |
|  | 2018  (n=5,852) | 2019  (n=9,502) | 2018  (n=4,946) | 2019  (n=6,431) | 2018  (n=4,885) | 2019  (n=12,503) |
| **Sex at birth** |  |  |  |  |  |  |
| Female | 48.0 (3306) | 49.1 (5789) | 48.7 (2971) | 48.8 (4643) | 50.1 (3231) | 49.5 (9257) |
| Male | 52.0 (2546) | 50.9 (3713) | 51.4 (1975) | 51.2 (1788) | 49.9 (1654) | 50.6 (3246) |
|  |  |  |  |  |  |  |
| **Age group** |  |  |  |  |  |  |
| 16-25 | 13.1 (581) | 14.4 (1177) | 12.7 (713) | 15.0 (996) | 13.3 (375) | 16.4 (1650) |
| 26-35 | 23.9 (901) | 24.5 (2030) | 21.3 (648) | 23.8 (1570) | 25.3 (853) | 24.3 (2996) |
| 36-45 | 19.6 (862) | 20.7 (2005) | 19.3 (754) | 20.6 (1350) | 16.9 (810) | 20.3 (2502) |
| 46-55 | 21.5 (1294) | 19.8 (1911) | 23.2 (1105) | 20.4 (1116) | 22.3 (1040) | 19.4 (2215) |
| 56-65 | 21.8 (2214) | 20.7 (2379) | 23.4 (1726) | 20.4 (1399) | 22.2 (1807) | 19.5 (3140) |
|  |  |  |  |  |  |  |
| **Ethnicity/Race** |  |  |  |  |  |  |
| White | 82.6 (5030) | 77.6 (7926) | 72.0 (4036) | 76.7 (5167) | 68.7 (3992) | 78.6 (9992) |
| Mixed/Other | 17.4 (822) | 22.4 (1876) | 28.0 (910) | 23.3 (1264) | 31.3 (893) | 21.4 (2511) |
|  |  |  |  |  |  |  |
| **Highest level of Education** |  |  |  |  |  |  |
| Less than high school | 12.9 (431) | 14.2 (694) | 9.5 (495) | 8.9 (569) | 7.6 (162) | 4.4 (462) |
| High school diploma | 27.7 (918) | 27.4 (1602) | 20.6 (870) | 23.7 (1512) | 17.3 (704) | 21.2 (2401) |
| Some college or technical vocation | 37.3 (2768) | 35.8 (4343) | 45.1 (1797) | 40.0 (2538) | 48.5 (1887) | 45.4 (5455) |
| Bachelor’s degree or higher | 22.0 (1714) | 22.7 (2784) | 24.8 (1776) | 27.4 (1799) | 26.5 (2127) | 29.1 (4141) |
|  |  |  |  |  |  |  |
| **Income adequacy** |  |  |  |  |  |  |
| Very difficult/Difficult | 31.6 (1761) | 36.3 (3231) | 33.3 (1601) | 35.5 (2464) | 31.2 (1417) | 36.6 (4708) |
| Neither easy nor difficult | 36.1 (2085) | 34.6 (3212) | 32.1 (1544) | 33.1 (2035) | 33.9 (1610) | 33.3 (4004) |
| Easy/Very Easy | 32.4 (1915) | 29.2 (2842) | 34.6 (1757) | 31.4 (1825) | 35.0 (1799) | 30.1 (3506) |
|  |  |  |  |  |  |  |
| **Cannabis use frequency** |  |  |  |  |  |  |
| More than 12 months ago | 51.3 (3439) | 43.1 (4433) | 56.5 (3027) | 51.0 (3323) | 44.3 (2541) | 43.0 (5621) |
| Past 12-month consumer | 15.2 (850) | 18.3 (1795) | 12.6 (629) | 13.1 (894) | 15.2 (749) | 14.7 (1922) |
| Monthly consumer | 8.6 (407) | 11.3 (978) | 9.6 (409) | 9.8 (596) | 11.0 (432) | 9.2 (1090) |
| Weekly consumer | 9.2 (407) | 9.1 (803) | 7.7 (322) | 7.6 (454) | 11.1 (388) | 9.3 (1078) |
| Daily/almost daily consumer | 15.7 (749) | 18.2 (1493) | 13.6 (559) | 18.6 (1164) | 18.3 (775) | 23.9 (2792) |
|  |  |  |  |  |  |  |

Weighted % and unweighted n. Difference in unweighted sample sizes are due to missing data in highest level of education in Canada (n=100) and the US (n=70); and income adequacy in Canada (n=308); and the US (n=495).

**Online Supplemental Material 3: Self-reported cannabis characteristics of consumers who had ever used cannabis for headaches or pain (n=15,092)**

|  | Canada  % (n) | | US ‘illegal’ states  % (n) | | US ‘legal’ states  % (n) | |
| --- | --- | --- | --- | --- | --- | --- |
|  | 2018  (n=1,485) | 2019  (n=2,883) | 2018  (n=1,264) | 2019  (n=2,427) | 2018  (n=1,647) | 2019  (n=5,386) |
|  |  |  |  |  |  |  |
| **Cannabis use frequency** |  |  |  |  |  |  |
| More than 12 months ago | 17.0 (271) | 18.7 (519) | 25.0 (319) | 30.2 (692) | 19.3 (285) | 22.6 (1176) |
| Past 12-month consumer | 13.2 (232) | 16.1 (515) | 14.5 (201) | 13.1 (353) | 15.2 (312) | 14.2 (833) |
| Monthly consumer | 14.4 (203) | 14.7 (423) | 15.4 (195) | 13.5 (322) | 14.6 (237) | 11.6 (650) |
| Weekly consumer | 16.2 (223) | 14.3 (436) | 15.5 (175) | 10.9 (262) | 16.4 (238) | 13.4 (683) |
| Daily/almost daily consumer | 39.1 (556) | 36.3 (990) | 29.8 (374) | 32.3 (798) | 34.5 (575) | 38.3 (2044) |
|  |  |  |  |  |  |  |
| **Problematic cannabis use***^ǂ^ |  |  |  |  |  |  |
| Low risk | 8.4 (123) | 12.1 (313) | 7.1 (78) | 9.9 (176) | 8.5 (187) | 11.0 (507) |
| Moderate risk | 90.3 (972) | 85.1 (1789) | 91.0 (789) | 86.4 (1394) | 89.9 (1078) | 86.8 (3292) |
| High risk | 1.3 (14) | 2.7 (42) | 2.0 (12) | 3.7 (33) | 1.6 (10) | 2.2 (59) |
|  |  |  |  |  |  |  |
| **Do you consider yourself to be addicted to cannabis?*** |  |  |  |  |  |  |
| Not at all | 64.7 (890) | 66.0 (1636) | 66.8 (668) | 70.5 (1260) | 70.0 (1044) | 70.5 (3109) |
| A little | 26.8 (242) | 22.5 (496) | 24.2 (204) | 18.3 (304) | 21.4 (232) | 20.7 (748) |
| Very | 6.6 (55) | 9.4 (167) | 7.7 (58) | 9.0 (124) | 7.0 (60) | 6.3 (217) |
| Don’t know | 1.9 (23) | 2.1 (58) | 1.3 (14) | 2.3 (41) | 1.6 (21) | 2.6 (117) |
|  |  |  |  |  |  |  |

*Asked among past 12-month cannabis consumers

ǂ Problematic cannabis use: Participants were asked six questions from the World Health Organization’s (WHO) Alcohol, Smoking, and Substance Involvement Screening Test (ASSIST) tool for problematic cannabis use: 1) “In the past 3 months, how often have you used marijuana?” (Never/Once or twice/Monthly/Weekly/Daily or almost daily); 2) “During the past 3 months, how often have you had a strong desire or urge to use marijuana?” (Never/Once or twice/Monthly/Weekly/Daily or almost daily); 3) “During the past 3 months, how often has your use of marijuana led to health, social, legal, or financial problems?” (Never/Once or twice/Monthly/Weekly/Daily or almost daily); 4) “During the past 3 months, how often have you failed to do what was normally expected of you because of your use of marijuana?” (Never/Once or twice/Monthly/Weekly/Daily or almost daily); 5) “Has a friend or relative or anyone else ever expressed concern about your use of marijuana?” (No, never/Yes, in the past 3 months/Yes, but not in the past 3 months); and 6) “Have you ever tried and failed to control, cut down, or stop using marijuana?” (No, never/Yes, in the past 3 months/Yes, but not in the past 3 months). Answers were categorized into “Low risk”, “Moderate risk”, and “High risk” according to the WHO ASSIST tool.

World Health Organization. WHO – ASSIST V3.0. 2010. Available at: <https://www.who.int/substance_abuse/activities/assist_v3_english.pdf> (accessed 13 September 2021).

**Online Supplemental Material 4: Weighted binary logistic regression analysis for the self-reported substitution of cannabis and pain medication in US states that have legalized recreational cannabis, states that have legalized medical cannabis only, and in states that have CBD-only laws or prohibited among respondents who had ever cannabis for headaches/migraines or pain**

|  | Have you ever used cannabis for pain relief, instead of using opioids or prescription pain medication?^a^  (n = 10,458)^a^  Yes  (vs. No) | |
| --- | --- | --- |
|  | Univariable  analysis  OR (95% CI) | Multivariable analysis  AOR (95% CI) |
| **Legal status of cannabis** |  |  |
| Prohibited or CBD-only | REF | REF |
| Medical only | 0.90 (0.73, 1.11) | 0.89 (0.72, 1.11) |
| Medical & Recreational | 1.08 (0.90, 1.30) | 1.04 (0.85, 1.26) |
|  |  |  |
| **Survey wave** |  |  |
| 2018 | - | REF |
| 2019 | - | 1.13 (0.96, 1.33) |
|  |  |  |
| **Cannabis use frequency** |  |  |
| Less than monthly^b^ | - | REF |
| Monthly/Weekly | - | **1.49 (1.26, 1.76)** |
| Daily | - | **2.50 (2.11, 2.97)** |
|  |  |  |
| **Sex at birth** |  |  |
| Female | - | 0.97 (0.84, 1.12) |
| Male | - | REF |
|  |  |  |
| **Age** | - |  |
| 16-25 | - | **0.79 (0.63, 0.99)** |
| 26-35 | - | 1.05 (0.86, 1.27) |
| 36-45 | - | 1.10 (0.90, 1.36) |
| 46-55 | - | 1.14 (0.91, 1.44) |
| 56-65 | - | REF |
|  |  |  |
| **Ethnicity/Race (vs. White)** |  |  |
| White | - | REF |
| Mixed/Other/Unstated | - | **0.78 (0.66, 0.92)** |
|  |  |  |
| **Highest level of Education** |  |  |
| Less than high school | - | REF |
| High school diploma | - | 0.96 (0.68, 1.34) |
| Some college or technical vocation | - | 0.85 (0.62, 1.18) |
| Bachelor’s degree or higher | - | **0.69 (0.50, 0.97)** |
|  |  |  |
| **Income adequacy** |  |  |
| Very difficult/Difficult | - | REF |
| Neither easy nor difficult | - | 0.86 (0.73, 1.01) |
| Very easy/Easy | - | **0.81 (0.68, 0.97)** |
|  |  |  |

^a^Asked among respondents who had ever consumed cannabis and ever consumed cannabis to manage or improve symptoms of headaches/migraines or pain.

^b^Includes respondents who have ever consumed cannabis and those who have consumed in the past 12-months but not monthly.
